# Characteristics of dihydroflavonol 4-reductase gene promoters from different leaf colored *Malus* crabapple cultivars

**DOI:** 10.1038/hortres.2017.70

**Published:** 2017-12-13

**Authors:** Ji Tian, Meng-chen Chen, Jie Zhang, Ke-ting Li, Ting-ting Song, Xi Zhang, Yun-cong Yao

**Affiliations:** 1 Department of Plant Science and Technology, Beijing University of Agriculture, Beijing 102206, China; 2 Beijing Collaborative Innovation Center for Eco-Environmental Improvement with Forestry and Fruit Trees, Beijing 102206, China

## Abstract

Anthocyanins are secondary metabolites in land plants that contribute to the colors of leaves and flowers, and are nutritionally valuable components of the human diet. The *DFR* gene plays an important role in the anthocyanin biosynthetic pathway. In this study, we investigated the regulation of *DFR* expression and in different *Malus* crabapple cultivars that show distinct patterns of leaf coloration, and how it influences leaf anthocyanin accumulation and coloration. Specifically, we studied the ever-red leaved cultivar ‘Royalty’, the ever-green leaved cultivar ‘Flame’ and the spring-red leaved cultivar ‘Radiant’. RT-PCR analysis showed that the expression of *McDFR1* correlated with the expression of a MYB transcription factor, *McMYB10*, and with anthocyanin accumulation. We isolated five *McDFR1* promoter fragments from the three cultivars and identified four different fragments (F1–4) that were present either in several cultivars, or only in one. Yeast one-hybrid and electrophoretic mobility shift assay analyses showed that McMYB10 could bind to all the *McDFR1* promoters, except *McDFR1-Ra2*. The F1, F2 and F3 fragments did not affect McMYB10 binding to the *McDFR1* promoters; however, we found evidence that the F4 fragment suppressed binding, and that the MYBGAHV amino-acid sequence maybe an important *cis*-element for McMYB10 protein binding. This information has potential value for strategies to modify plant color through genetic transformation.

## Introduction

Anthocyanins comprise a class of water-soluble flavonoids pigments in plants that contribute to the color of flowers, fruit, stems and leaves.^1,2^ They also function in vegetative tissues that provide protection against UV and high light irradiation, as antioxidants to scavenge reactive oxygen species (ROS), and as antimicrobial agents during defense responses.^3–6^ Anthocyanins are also potentially beneficial components of the human diet,^6,7^ and they can act as antioxidants, are anti-carcinogenic,^8,9^ anti-inflammatory^10^ and may help support both diabetes prevention and treatment,^11^ and heart health.^12^

**Figure 1:**
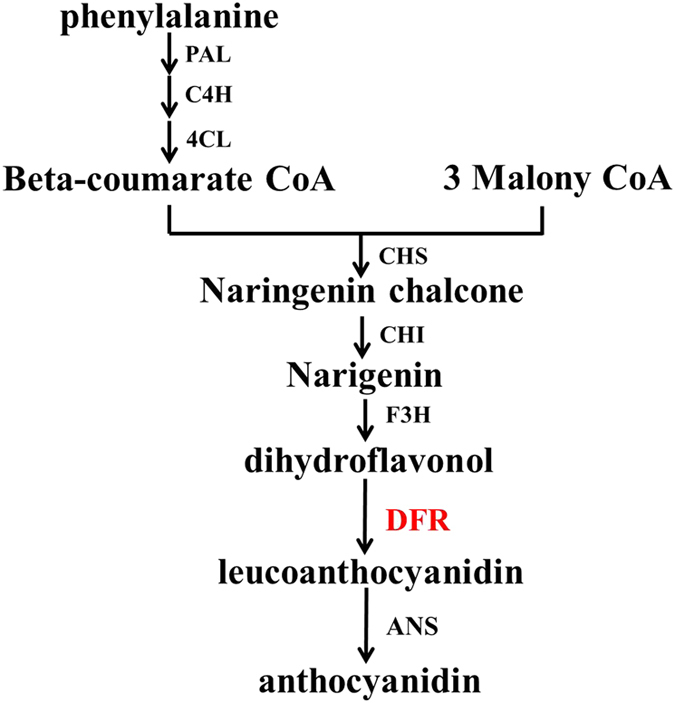
The flavonoid pathway leading to the synthesis of anthocyanins and flavonols. ANS, anthocyanidin synthase; CHI, chalcone isomerase; C4H, cinnamate 4-hydroxylase; CHS, chalcone synthase; DFR, dihydroflavonol reductase; F3H, flavonoid 3-hydroxylase; PAL, phenylalanine ammonia lyase; 4CL, 4-coumarate CoA ligase.

Over the past few decades, the anthocyanin biosynthetic pathway has been well characterized in plants, such as arabidopsis (*Arabidopsis thaliana*), petunia (*Petunia hybrida*), maize (*Zea mays*), snapdragon (*Antirrhinum majus*) and apple (*Malus domestica*).^13–17^ Most of the genes encoding enzymes responsible for each step in the flavonoid biosynthetic pathway have been identified,^18^ and it has been established that dihydroflavonol 4-reductase (DFR) plays a key role in the formation of common and condensed anthocyanins (Figure 1).^18^ DFR is one of the rate-limiting enzymes that catalyzes the production of flavan-3,4-diols (leucoanthocyanidins) via the reduction of three colorless dihydroflavonols: dihydrokaempferol (DHK), dihydroquercetin (DHQ); and dihydromyricetin (DHM). These three compounds are also intermediates in flavonol biosynthesis through the flavonol synthase reaction, using NADPH as a cofactor.^19,20^

A number of *DFR* genes have been characterized from a wide range of plant species, including *Camellia sinensis* (tea),^21^ *Medicago truncatula* (Medicago),^22^ *Ipomoea batatas Lam* (sweet potato),^23^ *Ginkgo biloba* (ginkgo)^24^ and *Populus trichocarpa*.^25^ It has also been shown that overexpression of different *DFR* genes in tobacco (*Nicotiana benthamiana*) flowers promotes anthocyanin biosynthesis, corresponding to an increase in red pigmentation.^26^ In addition, suppressing *IbDFR* in sweet potato led to a decrease in anthocyanin accumulation and reduced the tolerance to abiotic stress.^23^ *DFR* not only regulates levels of anthocyanin, but also shows substrate specificity, resulting in the accumulation of different types of anthocyanins. For example, petunia (*Petunia hybrida (J. D. Hooker) Vilmorin*) and cymbidium (*Cymbidium faberi Rolfe*) lack varieties with brick red/orange colored flowers due to a lack of pelargonidin-based anthocyanins because DFRs do not utilize dihydrokaempferol as a substrate.^27,28^ Furthermore, overexpressing *MtDFR* in rice also changed several metabolites except anthocyanins, including the concentrations of amino acids, sugars and metals.^29^ Altered the expression of DFR also affect the expression of other anthocyanin biosynthesis gene. The upregulation of *McDFR* expression in crabapple leaves or apple peels was accompanied by a proportional increase in some of the genes involved in anthocyanin biosynthesis (*McCHS*, *McCHI*, *McF3H*, *McF3’H*, *McDFR*, *McANS* and *McUFGT*).^30^ In addition, overexpression of *RrDFR1* (*Rosa rugosa*) and *PhDFR1* (*Petunia hybrida*) genes in tobacco displayed downregulation of the endogenous *NtFLS* gene, and the promotion of anthocyanin synthesis. The relative expression levels of *NtCHS* and *NtFLS* were significantly downregulated and *NtANS* genes was upregulated in *DFR* transgenic lines.^26^

In plants, MYB (v-myb avian myeloblastosis viral oncogene homolog), bHLH (basic-helix-loop-helix) and WD40 (Trp-Asp 40) transcription factors (TFs) often form MYB-bHLH-WD40 (MBW) complexes and play important roles in secondary metabolism, development, signal transduction and disease resistance. It has been shown that several MYB TFs are involved in positively regulating the expression of *DFR*.^31^ In *A. thaliana*, the expression of the *MYB75/PAP1* gene is induced by light and precedes the expression of both *MYB90/PAP2* and several structural genes (*CHS*, *DFR*, *F3H* and *LDOX*).^32^ Moreover, the apple TFs *MdMYB10* and *MdMYB1* control the red pigmentation in the fruit flesh and skin, respectively,^33^ and the corresponding proteins, MdMYB10 and MdMYB1, can form complexes with bHLH genes to *trans*-activate the *DFR* promoter and promote anthocyanin accumulation.^34^ miR156 targets, SPL9, negatively regulates anthocyanin accumulation by directly preventing expression of anthocyanin biosynthetic genes DFR through destabilization of a MYB-bHLH-WD40 (PAP1, TT8 and TTG1) transcriptional activation complex.^32^ In contrast, several MYB transcription factors also have been proved that they can suppress the expression of *DFR*. *AtMYBL2* interacts with *TT8* (TRANSPARENT TESTA 8) to reduce anthocyanin biosynthesis by suppressing the expression of *DFR*.^33^ The expression of the *AtMYB7* gene is induced by salt stress, and represses several flavonoid pathway genes, including *DFR* and *UGT*.^35^ Furthermore, MYBCORE, MYBGAHV *cis*- regulatory elements have been proved that involved in MYB transcription factors binding to several promoters to regulate gene expression, such as MYB101 regulates fertilization in *Arabidopsis thaliana* by binding to the MYBGAHV elements in the promoters of downstream genes, and MYB5 and TT2 regulates proanthocyanins accumulation in Arabidopsis seed coat by binding to the MYBCORE elements in the promoter of *DFR*.^36,37^

Structural differences in promoter regions contribute to the difference in expression of the target genes regulated by TFs.^38^ In apple, a TATA-box insertion in the IRT1 promoter is responsible for increasing Fe uptake, and its presence correlates with an increase in transcriptional activation by specific binding of the TF, IID.^39^ Meanwhile, promoter structure also affect anthocyanins-related gene expression in plants. Subsequent analysis showed that a rearrangement of the 23-bp sequences in the promoter regions of *MdMYB10* and *McMYB10* was responsible for the difference in gene expression between the white- and red-fleshed apples and red-leaf color crabapples.^33^ Specifically, the R1: MdMYB10 (McMYB10) promoter has a single MdMYB10 (McMYB10) binding motif, and is only present in white-fleshed apples, while the R6: MdMYB10 (McMYB10) promoter, which is present in red-fleshed apples, has five additional tandem repeats of the MdMYB10 (McMYB10) binding motif. Our studies in *Malus *crabapple have shown that a 743-bp fragment missing of *McCHS* promoter was found in ever-green leaf color crabapple cultivar, and the missing sequence contained two MYBPLANT elements and three MYBCORE elements that are essential for MYB transcription factor binding and inducing anthocyanin biosynthesis. So, we conclude that different structures in the chalcone synthase (*McCHS*) promoter affect the binding of MYB TFs and the expression of *McCHS*, and lead to the accumulation of different anthocyanins in crabapple cultivars with different leaf colors.^40^ However, little is known about the basis for the differences in DFR expression caused by MYB TFs.

*Malus* crabapples are collectively an economically important germplasm resource for ornamental plants, providing numerous landscape species. They also exhibit stress resistance and provide valuable research material to investigate the mechanisms of anthocyanin accumulation and color formation, reflected by the diversity of leaf, flower and fruit coloration.^41^ In this current study, the expression analysis showed that *McDFR1* may play an important role in anthocyanins biosynthesis in crabapple leaves, hence we examined differences in the *McDFR1* promoter sequences and functions in three different types of *Malus* crabapple: *Malus* cv. ‘Flame’, *Malus* cv. ‘Royalty’ and *Malus* cv. ‘Radiant’. We investigated whether variations in the *DFR1* promoter may affect the expression of *DFR1* in different colored leaf crabapple cultivars and provide evidence that the *McDFR1* promoter has potential applications to improve plant color via genetic transformation approaches.

## Materials and methods

### Material and growth conditions

Three crabapple cultivars were used in this study: ‘Royalty’, an ever-red leaved cultivar; ‘Flame’, an ever-green leaved cultivar; and ‘Radiant’, which has red young leaves that turn green as they mature. Eight-year-old crabapple trees grafted onto a *Malus* ‘Balenghaitang’ stock were grown in the Crabapple Germplasm Resources Nursery, Beijing University of Agriculture (40.l″ north latitude, 116.6″ longitude). In mid-late April (18–22 °C) and on sunny days, we selected six trees of each cultivar that showed similar growth and collected leaf samples from annual branches growing in all four compass directions on the outer edge of each canopy. Leaves of ‘Royalty’, ‘Radiant’ and ‘Flame’ were collected at 10 developmental stages (3, 6, 9, 12, 15, 18, 21, 24, 27 and 30 days after budding). Branches to be used for the experiments were marked before budding. All samples were immediately frozen in liquid nitrogen and stored at −80 °C.

### High-performance liquid chromatography analysis

The crabapple leaves (approximately 0.8–1.0 g fresh weight) were extracted with 10 mL extraction solution (methanol:water:formic acid:trifluoroacetic acid=70:27:2:1),^42^ at 4 °C in the dark for 72 h, with shaking every 6 h. The homogenate was then filtered through sheets of qualitative filter paper and the filtrate was then passed through a 0.22 μL reinforced nylon membrane filter (Millipore, Billerica, MA, USA). The resulting sample was subjected to high-performance liquid chromatography (HPLC) using an HPLC1100-DAD system (Agilent Technologies, Waldbronn, Germany), with trifluoroacetic acid: formic acid: water (0.1:2:97.9) as mobile phase A and trifluoroacetic acid: formic acid: acetonitrile: water (0.1:2:48:49.9) as mobile phase B.^43^ The gradients used were as follows: 0 min, 30% B; 10 min, 40% B; 50 min, 55% B; 70 min, 60% B; 80 min, 30% B. Detection of anthocyanins was performed at 520 nm. All samples were analyzed in biological triplicate.

### Cloning and analysis of the *McDFR1* promoters

To analyze the differences in *McDFR1* promoter sequences of the three different leaf colored crabapple cultivars, genomic DNA was isolated from ‘Royalty’, ‘Radiant’ and ‘Flame’ leaves using the Plant Genomic DNA Kit (TIANGEN BIOTECH CO., LTD, Beijing, China). The 5′-upstream sequences were amplified by hi-TAIL PCR^44^ the primers are shown in Supplementary Table S1. The primers for hi-TAIL PCR (high-efficient thermal asymmetric interlaced PCR) were designed based on the *McDFRs* cDNA sequence (GenBank Accession: FJ817487, AF117268). The upstream-flanking sequence of *McDFR1* was isolated using hi-TAIL PCR. The hi-TAIL PCR is a method to isolate upstream (promoters) and downstream sequences of the known coding sequences. Long (33–34 nucleotides) arbitrary degenerate (LAD) primer with a higher degree (2304 or 6912 folds) of degeneracy is used to create primer-binding sites efficiently along the unknown target sequences and used for the first amplification. An additional sequence (AC1, 18 nucleotides with *T*_m_=58 °C, used for the second and third amplification) identical to the 5′ part of the LAD primer is tagged to the 5′ end of a long primer (*T*_m_>68 °C) specific to the known sequence. The forward special primers (McDFR1-promoter1, McDFR1-promoter2, and McDFR1-promoter3; Supplementary Table S1) used in three reactions were based on the *McDFR1* coding sequence (GenBank Accession: FJ817487). All PCR products were sub-cloned into the pGEM T-Easy Vector (Promega, Madison, WI, USA) and transformed into *Escherichia* *coli* DH5α cells and sequenced. The *cis*-elements were analyzed using the PLACE database (http://www.dna.affrc.go.jp/PLACE/).

### RNA extraction and semi-quantitative RT-PCR

Total RNA was extracted from crabapple leaves using the RNA Extract kit (Aidlab, Beijing, China) according to the manufacturer’s instructions. DNase I (TaKara, Ohtsu, Japan) was added to remove genomic DNA, and the 1 μg RNA samples were subjected to cDNA synthesis using the Access RT-PCR System (Promega), according to the manufacturer’s instructions.

Semi-quantitative RT-PCR was carried out in 20 μL reactions with 2 μL of 10× diluted cDNA template, and *McActin* was used as the internal control.^45^ transcription system (Promega) PCR amplification system with the following conditions: initial denaturation at 95 °C for 3 min, followed by 20 or 29 cycles (29 cycles for *McDFR1*, *McDFR1*, *McMYB10* and 20 cycles for *McActin*) of 95 °C for 10 s, 60 °C (for *McDFR1*, *McDFR1*, *McMYB10* and *McActin*) for 30 s and extension at 72 °C for 30 s and final denaturation was at 72 °C for 5 min, maintain at 4 °C. Specific primers for semi-quantitative RT-PCR analysis were designed using primer 5 software and are listed in Supplementary Table S1.^46^

The expression levels of *McDFR1*, *McDFR2* and *McMYB10* in crabapple were analyzed using qRT-PCR and the SYBR Green qPCR Mix (TaKaRa) and the Bio-Rad CFX96 Real-Time PCR System (Bio-Rad, Hercules, CA, USA), according to the manufacturers’ instructions. The PCR primers were designed using NCBI Primer BLAST and are listed in Supplementary Table S1. qRT-PCR analysis was carried out in a total volume of 20 μL containing 9 μL of 2×SYBR Green qPCR Mix (TaKaRa), 0.1 μM specific primers (each), and 100 ng of template cDNA. The reaction mixtures were heated to 95 °C for 30 s, followed by 39 cycles at 95 °C for 10 s, 50–59 °C for 15 s and 72 °C for 30 s. A melting curve was generated for each sample at the end of each run to ensure the purity of the amplified products. The transcript levels were normalized using the *Malus *18S ribosomal RNA gene (DQ341382, for apple and crabapple) as the internal controls and calculated using the 2^(−ΔΔCt)^ analysis method.^46^

### Yeast one-hybrid assay

A yeast one-hybrid system was used to assay the relationship between the McMYB10 protein and the *McDFR1* promoters from the three *Malus* crabapple cultivars.^47^ As the effector construct, the open reading frame of *McMYB10* was cloned into the *BamH*I and *Sal*I sites of the pJG4-5 vector (Clontech, Palo Alto, CA, USA) under the control of the galactokinase 1 (GAL1) promoter. The *McDFR1* promoter sequences were inserted upstream of the *LacZ* reporter gene in the pLacZi vector. The effector and reporter or control constructs were transformed into competent cells of the yeast strain EGY48, resulting in the following yeast strains: pJG4-5-McMYB10/ pLacZi-promoters of *McDFR1*; pJG4-5/ pLacZi-promoters of *McDFR1*; pJG4-5-McMYB10/pLacZi; and pJG4-5/ pLacZi. The yeast cells were selected on synthetic drop-out media lacking tryptophan and uracil, and positive colonies were spotted onto glucose plates (2%) containing X-gal at 28 °C for 2 days and examined for blue color development.^45^

### Electrophoretic mobility shift assay

The *McMYB10* open reading frame sequence was cloned into the pMAL-C2X expression vector and transformed into Rosetta (DE3) *E. coli* competent cells.^48^ The maltose-binding protein (MBP)-tag encoded in the pMAL-C2X vector was used to facilitate purification of the recombinant protein. Isopropyl β-d-1-thiogalactopyranoside (0.3 mM) was added to the cultures to induce *McMYB10* expression in a cell culture shaking at 170 r.p.m. for 6 h at 28 °C. The recombinant protein was purified with the One-Stop MBP-Tagged Protein Miniprep Pack (BioLab. Co. Ltd, Beijing, China). Electrophoretic mobility shift assay (EMSA) reactions were prepared according to the manufacturer’s protocol (LightShif Chemi luminescent EMSA Kit; Termo Fisher Scientifc, Waltham, MA, USA). The probes were labeled by annealing biotin-labeled oligonucleotides. The same 100×unlabeled DNA fragment was used as a competitor in the assay. The oligonucleotides used for EMSA are listed in Supplementary Table S2. Approximately 10 μg of purified McMYB10 recombinant protein was used for each EMSA reaction.

## Results

### *McDFR* expression and anthocyanin accumulation during leaf development

**Figure 2:**
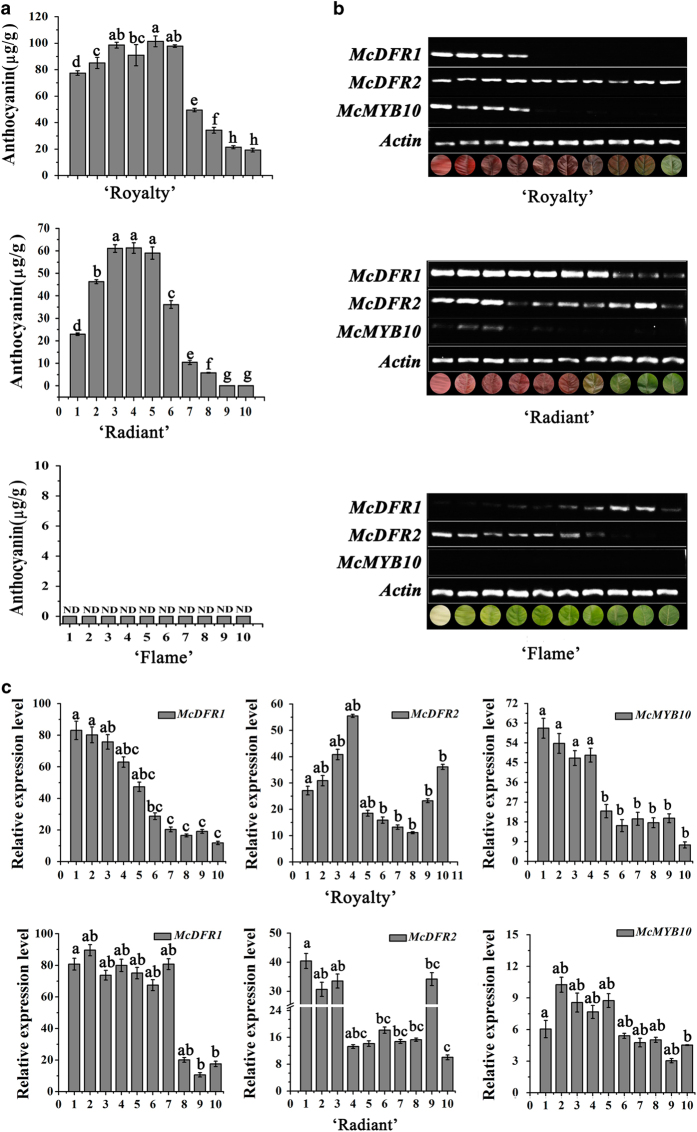

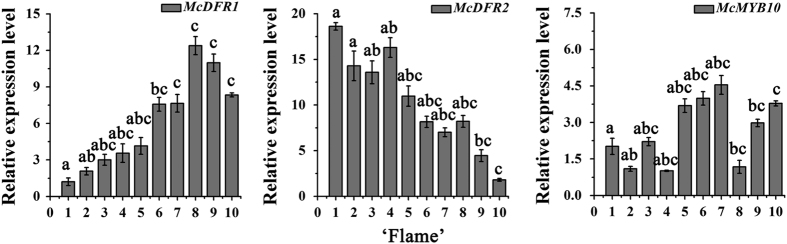
Analysis of flavonoid accumulation and the expression profiles of *McDFR* genes and *McMYB10* during 10 leaf developmental stages in the *Malus* crabapple ever-red cultivar ‘Royalty’, spring-red cultivar ‘Radiant’ and ever-green cultivar ‘Flame’. (**a**) The anthocyanin content in 10 leaf developmental stages of ‘Royalty’, ‘Radiant’ and ‘Flame’. (**b**) The expression of *McDFR1*, *McDFR2* and *McMYB10* analyzed by RT-PCR. (**c**) Real-time PCR was used to analyze *McDFR1*, *McDFR2*, *McMYB10* expression patterns in the leaves of ‘Royalty’, ‘Radiant’ and ‘Flame’. *Malus 18S* (DQ341382) was used as the reference gene. Error bars indicate the s.e.m.±s.e. of three replicate measurements. Different letters above the bars indicate significantly different values (*P*<0.05) calculated using one-way analysis of variance (ANOVA) followed by a Tukey’s multiple range test. ND, no detection. 1–10 represents stage 1–10 of leaf development stages. 1, 3 days after budding; 2, 6 days after budding; 3, 9 days after budding; 4, 12 days after budding; 5, 15 days after budding; 6, 18 days after budding; 7, 24 days after budding; 8, 21 days after budding; 9, 27 days after budding; 10, 30 days after budding.

*McDFR* is known to play an important role in anthocyanin biosynthesis.^49^ There are two DFR homologs in crabapple, and the full-length *McDFR* cDNAs of these two genes were cloned from cDNA libraries that from the leaf of ever-red leaf color crabapple cultivar ‘Royalty’, and named *McDFR1* and *McDFR2* (FJ817487, AF117268). To verify the relationship between the expression levels of the *McDFR* genes and anthocyanin accumulation, we examined anthocyanin accumulation and the expression profiles of the *McDFR* genes and *McMYB10* in 10 leaf developmental stages (3, 6, 9, 12, 15, 18, 21, 24, 27 and 30 days after budding) of the three crabapple cultivars by HPLC and RT-PCR, respectively (Figure 2).

We observed that in the ever-red ‘Royalty’ cultivar, the abundance of anthocyanins was relatively high in the first six developmental stages, before decreasing to low levels in the last four stages. Anthocyanin levels increased during the first five development stages and a gradual decrease was observed in the last five development stages in the spring-red crabapple cultivar ‘Radiant’, while they were almost undetectable in the ever-green ‘Flame’ cultivar.

RT-PCR analyses showed that the expression level of *McDFR1* were higher expressed in the first four and seven stages and gradually decreased in ‘Royalty’ and ‘Radiant’, respectively, but gradually increase in ‘Flame’ and highest in the stage of eight. The transcription of *McDFR2* gradually decrease in these three crabapple cultivars except stage 9 in ‘Radiant’. *McMYB10* was not detected in ‘Flame’ leaves and showed almost same expression trend with *McDFR1* in ‘Royalty’ and ‘Radiant’. We also performed qRT-PCR to quantified the expression distinction of *McDFR1*, *McDFR2* and *McMYB10* in these three cultivars, the results further confirmed that expression variations of these three genes in three crabapple cultivars. The expression level of *McDFR1* and *McMYB10* in leaf colored cultivars significantly higher than that in green-leaf crabapple cultivar ‘Flame’. Furthermore, the higher expression level of *McDFR2* in ‘Flame’ was observed, from which we inferred that *McDFR2* maybe involved in the biosynthesis of other flavonoid compounds than anthocyanins. Meanwhile, the correlation analysis showed the expression of *McDFR1* is closely related to the accumulation of anthocyanins and the transcription of *McMYB10* in crabapple leaves, and *McDFR2* only have 25.3% and 8.8% relativity with anthocyanins accumulation in ‘Royalty’ and ‘Radiant’ during leaf development, respectively (Supplementary Table S3).

Taken together, these data suggest that *McDFR1* and *McMYB10* expression is associated with anthocyanin accumulation in crabapple leaves and that *McMYB10* may be involved in the modulation of anthocyanin levels by regulating *McDFR1*. However, the mechanism by which *McMYB10* controls the expression of *McDFR1* is unclear.

### Sequence analysis of the *McDFR1* promoter and differences between the three *Malus* crabapple cultivars

Since the expression level of *McDFR1* is consistent with anthocyanins accumulation and the transcription of *McMYB10*, we speculated that *McDFR1* may play an important role in anthocyanins biosynthesis in crabapple leaves, hence we focused on the reason underlying this expression difference of *McDFR1* in ‘Royalty’, ‘Radiant’ and ‘Flame’. We demonstrate there are natural variations in the promoter region of the *McDFR1* gene. *McDFR1* promoter from ‘Royalty’ is homozygous (1590 bp, MF592793), named *McDFR1-R*; *McDFR1* promoters from ‘Flame’ are heterozygous, alleles of the ‘Flame’ *McDFR1* promoters were named *McDFR1-F1* and *McDFR1-F2* (F1, 1603 bp, MF592792; F2, 1445 bp, KT276932.1), respectively; *McDFR1* promoters from ‘Radiant’ are also heterozygous, *McDFR1* promoters were named *McDFR1-Ra1* and *McDFR1-Ra2* (Ra1, 1610 bp, MF592794; Ra2, 1693 bp, MF592795), respectively.

**Figure 3:**
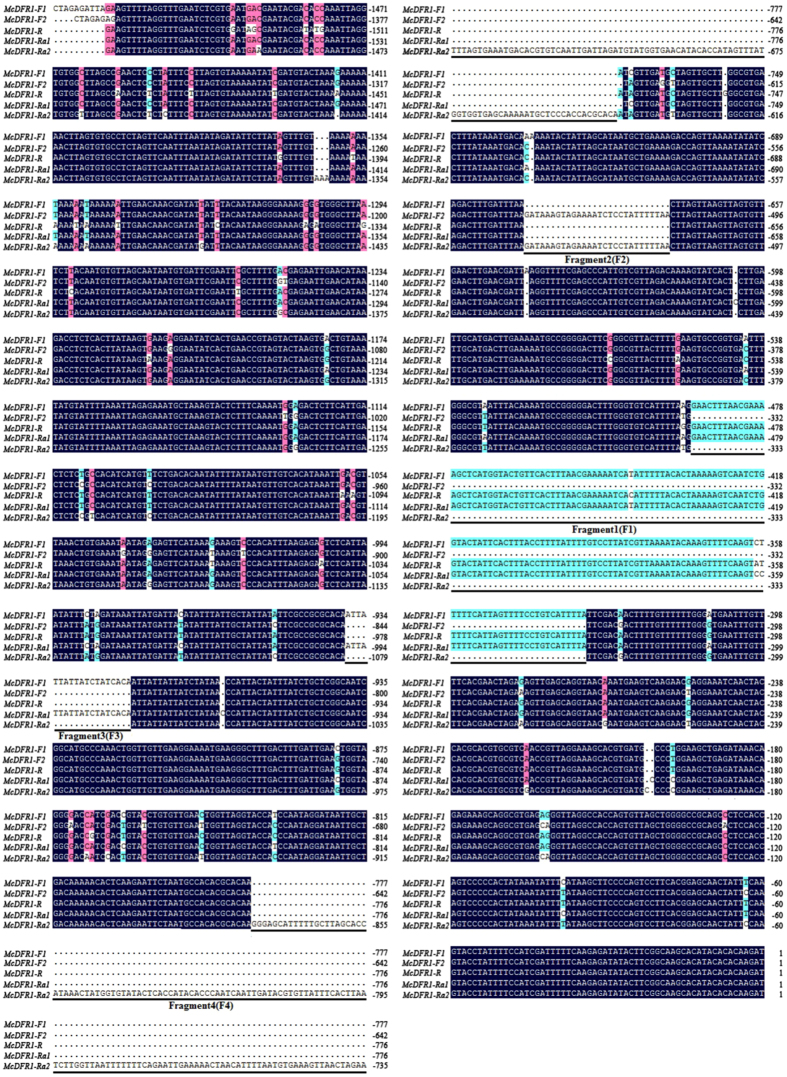
The sequence characteristics of the *McDFR1* promoter of ‘Flame’, ‘Royalty’ and ‘Radiant’. Alignment of the promoter sequences of *McDFR1*in three typical leaf colored crabapple cultivars. The *McDFR1* promoter from ‘Royalty’ was named *McDFR1-R*, the two *McDFR1* promoters from ‘Flame’ were named *McDFR1-F1* and *McDFR1-F2*, and the two *McDFR1* promoters from ‘Radiant’ were named *McDFR1-Ra1* and *McDFR1-Ra2*. We named the different fragments F1, F2, F3 and F4, respectively.

Among the five *McDFR1* promoter sequences, we found four different sequences that were either shared, or unique, and named them F1, F2, F3 and F4 (Figure 3). The F1 fragment (160 bp) was detected in the *McDFR1-F1*, *McDFR1-R* and *McDFR1-Ra1* promoters, the F2 fragment (28 bp) was found in the *McDFR1-F2* and *McDFR1-Ra2* promoters, and the F3 fragment (19 bp), an AT-rich fragment, was found in the *McDFR1-F1* and *McDFR1-Ra1* promoters. Interestingly, a 234 bp insertion was only observed in the *McDFR1-Ra2* promoter. We hypothesized that *McDFR1* promoter sequence diversity may influence the expression levels of *McDFR1*.

### *Cis*-element analysis of the *McDFR1* promoter

**Figure 4:**
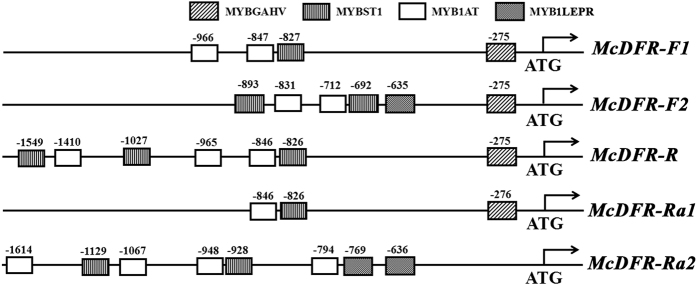
Schematic overview of *McDFR1-F1*, *McDFR1-F2*, *McDFR1-R*, *McDFR1-Ra1* and *McDFR1-Ra2* promoter features. Prediction of the *cis*-acting elements in the 1600-bp *McDFR1* promoter region of the three crabapple cultivars was performed using the PLACE and PlantCare databases.

To understand the mechanism of *McDFR1* transcriptional regulation by MYB TFs, we used the homologous *cis*-regulatory elements of the five *McDFR1* promoter sequences to search for known elements using the PLACE database,^50,51^ with a focus on putative MYB-binding sites. The MYB2AT, MYB2CONSENSUSAT, MYBATRD22, MYBCORE, MYBCOREATCYCB1 and MYBPZM elements, which are required for MYB TF binding, were present in almost the same positions in the five *McDFR1* promoters (Table 1). However, the number and the positions of MYB1AT, MYB1LEPR, MYBGAHV and MYBST1 varied. MYB1AT and MYBST1 were located 800 bp upstream of the ATG transcriptional start site and were present in various numbers, while MYB1LEPR were present 600 bp upstream from the ATG in the two promoters that did not contain F1 fragments. MYBGAHV was present in the two ‘Flame’ *McDFR1* promoters, the *McDFR1-R* promoter and the *McDFR1-Ra1* promoter, but not in the *McDFR1-Ra2* promoter, ~300 bp upstream of the ATG (Table 1 and Figure 4). We deduced that the MYBGAHV element may play a role in regulation of *McDFR1* expression by MYB TFs.

### Interaction of the *McDFR1* promoter with *McMYB10*

**Figure 5:**
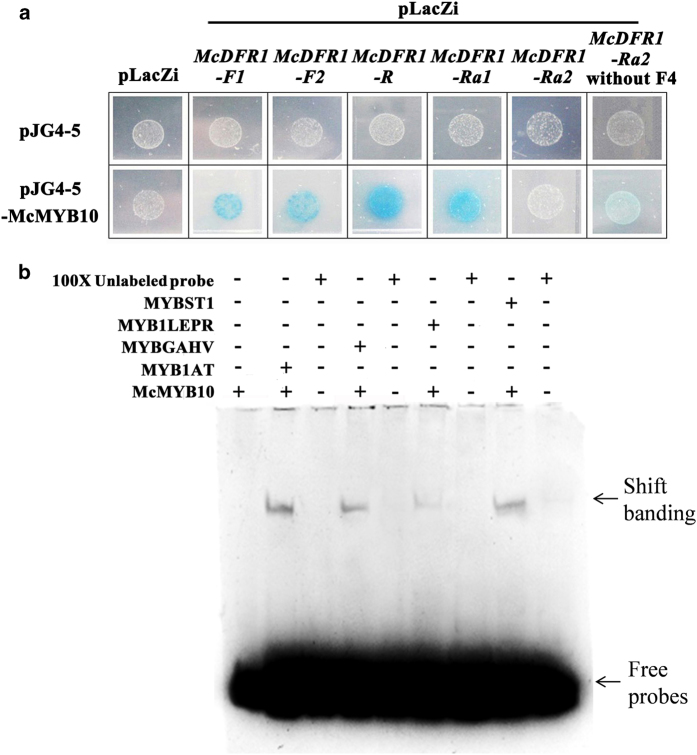
*Cis*-element binding ability and yeast one-hybrid assay of the *McDFR1* promoters with McMYB10. (**a**) Interaction of the McMYB10 protein with the *McDFR1-F1*, *McDFR1-F2*, *McDFR1-R*, *McDFR1-Ra1*, *McDFR1-*Ra2 and *McDFR1-Ra2 *(without F4) promoter regions, as revealed using yeast one-hybrid assays. The panel shows yeast cells containing distinct effector and reporter constructs grown on an SD/-Trp/-Ura medium plate. The interaction of McMYB10, fused to the GAL4 activation domain (pJG4-5-McMYB10), with LacZ driven by *McDFR* promoters (pLacZi-promoters of *McDFR1*) is shown in the bottom panel. Yeast transformed with pJG4-5-McMYB10/pLacZi, pJG4-5/pLacZi-*McDFR1* promoters and pJG4-5/pLacZi were used as controls. (**b**) Electrophoretic mobility shift assay (EMSA) of four the *cis*-elements, MYB1AT, MYBGAHV, MYB1LEPR, MYBST1, with McMYB10. 100×Unlabeled probe refers to the control of adding 100 times the concentration of a competing non-labeled specific probe. The black arrow indicates protein-DNA complexes, and the white arrow shows the positions of free probes. In lanes with competitor DNA, there was an excess of unlabeled probe.

To test the hypothesis that the F1, F2, F3 and F4 fragments affect the expression of *McDFR1*, which is regulated by *McMYB10* in crabapple, we evaluated McMYB10 binding to the *McDFR1-F1*, *McDFR1-F2*, *McDFR1-R*, *McDFR1-Ra1*, *McDFR1-Ra2* using the yeast one-hybrid assay (Figure 5a). *LacZ* activity was detected in yeast cells containing pJG4-5-McMYB10 together with pLacZi-*proMcDFR1-F1*, pLacZi-*proMcDFR1-F2*, pLacZi-*proMcDFR1-R*, pLacZi-*proMcDFR1-Ra1*, but not in yeast harboring pLacZi-*proMcDFR1-Ra2* together with pJG4-5-McMYB10, or in the control. Hence we hypothesized that the absence of MYBGAHV element or existence of F4 fragment might affect the activity of McMYB10 binding to the *McDFR1* promoter. To further investigate whether McMYB10 binds directly to the MYB1AT, MYB1LEPR, MYBGAHV and MYBST1 *cis*-elements, we performed an EMSA (Figure 5b). Biotin-labeled probes were designed to extend 10–20 bp on either side of the elements located nearest to the ATG transcriptional start site of each sequence. We found that McMYB10 bound to all four elements, and that this binding diminished gradually with 100× concentration of unlabeled probe. Furthermore, the binding of MYB1AT, MYBGAHV and MYBST1 to the McMYB10 protein was stronger than that of MYB1LEPR. Then to determine whether F4 fragment involved in McMYB10 binding to the *McDFR1* promoter, we generated pLacZi-*proMcDFR1-Ra2* (without F4), which were based on the native *Ra2* promoter sequences, but without F4 fragment. The yeast cells containing pJG4-5-McMYB10 and pLacZi-*proMcDFR1-Ra2* (without F4) showed a light blue color.

From these results we concluded that the F4 fragment may suppress the binding of McMYB10 to the *McDFR1*-*Ra2* promoter, and that the absence of MYBGAHV from the *McDFR1-Ra2* promoter maybe the main reason why McMYB10 bound only weakly to the *McDFR1-Ra2* (without F4) promoter. The F1, F2 and F3 *McDFR1* promoter fragments did not affect McMYB10 binding to the *McDFR1* promoters.

## Discussion

There is considerable interest in the breeding of ornamental plants with colored leaves, especially those with red/purple leaves, which is mainly due to the accumulation of anthocyanins.^52–54^ However, the molecular mechanism of red color formation in leaves is still unclear. DFR is a key enzyme in the catalysis of the stereo-specific reduction of dihydroflavonols to leucoanthocyanidins, which uses NADPH as a cofactor and is located in a key branch point in the anthocyanin biosynthetic pathway.^55^ The expression level variations of *DFR* determine the color of leaves, flowers and fruits in many plants,^56–58^ and given the central role of DFR in anthocyanin accumulation, we investigated the possible regulatory mechanism by which MYB TFs control *DFR* gene expression in crabapple leaves.

HPLC analysis and transcript quantification suggested that red leaves were associated with higher anthocyanin accumulation, which was consistent with increased transcript levels of *McDFR* and *McMYB10* (Figure 2). Several studies have shown that *MYB10* can activate the expression of *DFR* and promote anthocyanin accumulation. In *Arabidopsis thaliana*, *AtDFR* is a target gene of *MYB75/PAP1* and its expression can be enhanced by an elevated expression of *MYB75*.^59^ *MdMYB10* and *MdMYB1* have been shown to be able to bind the *DFR* promoter and promote anthocyanin accumulation in apple skin.^34^ Therefore, we hypothesized that *McDFR* maybe a *McMYB10* target and that high expression of *McDFR* might result in red leaf coloration. To address this, we examined the expression level of two *McDFR* genes and *McMYB10* in three crabapple cultivars and the results showed that *McDFR1* have positively relationship with the expression of *McMYB10* and anthocyanins accumulation in crabapple leaves. Hence, we focused on the regulation mechanism of *McDFR1* in crabapple. We cloned five *McDFR1* promoter fragments. Interestingly, low expression of the *McDFR* genes and low expression of *McMYB10* were observed in the ever-green leaf colored cultivar ‘Flame’ (Figure 2a). We speculate that *McDFR* genes is responsible for the accumulation of flavonoid products other than anthocyanins in ‘Flame’. Moreover, since LacZ activity driven by the *McDFR1-F1*, *McDFR1-F2* and *McDFR1-R* promoters in the yeast one-hybrid assay (Figure 5a) was detectable, we hypothesize that there was no anthocyanin accumulation in the green leaves as a consequence of low *MYB10* expression, and the low level of expression of genes in the anthocyanin biosynthetic pathway.

In a previous study, 1 kb insertion in the promoter that increases the expression of citrate transporter gene *HvAACT1* in several Al-tolerant barley cultivars.^60^ In addition, a deletion in the promoter of a mitochondrial molybdenum transporter gene (*MOT1*) in *Arabidopsis* is associated with reduced gene expression and low molybdenum (Mo) levels in the shoot.^61^ The same nucleotide polymorphisms were also appeared in anthocyanins-related genes. An extra fragment (255 bp) in the promoter of *TfF3′H1* in reddish flower tulip sport *Tulipa fosteriana* hampered the accumulation of cyanidin anthocyanins through the reduction of *TfF3′H1* transcription.^62^ A 23 bp repeat motif in the upstream regulatory region of *MYB10* alleles was found only in red-fleshed apples and red leaved crabapples.^33,63^ Moreover, this promoter allele was shown to be responsible for the increased accumulation of anthocyanins, and the number of repeat units correlated with an increase in *trans*-activation by the MYB10 protein.^33^ A 743 bp deletion fragment in the *McCHS* gene promoter in the ever-green leaf colored crabapple cultivar ‘Flame’ would lead to only a few MYB TFs binding to the promoter sequence and reduce *McCHS* expression, reflecting a direct relationship with anthocyanin content and leaf color.^33^ This suggests structural differences in the promoter are an important factor in determining the gene expression variations in the different cultivars. To determine the expression pattern of *McDFR1* in different crabapple cultivars, we isolated five *McDFR1* promoter fragments from three typical leaf colored cultivars (Figure 3). The results showed that the promoters of *McDFR1* contain many MYB-binding *cis-*elements, consistent with *McDFR1* being regulated by MYB TFs. The yeast one-hybrid results showed that McMYB10 could not bind to the *McDFR1-Ra2* promoter, but when the F4 fragment was deleted in the *McDFR1-Ra2* promoter, binding was partially restored compared with the original *McDFR1-Ra2* promoter (Figure 5a). Furthermore, EMSA analyses suggested that McMYB10 binds to the MYB1AT, MYBGAHV, MYB1LEPR and MYBST1 *cis*-elements in the *McDFR1* promoters (Figure 5b), and we also found that the MYBGAHV element that was not present in *McDFR1-Ra2* and *McDFR1-Ra2* (without F4) affected McMYB10 binding to the *DFR1* promoter to increase *DFR* expression. We concluded that the F4 fragment in the *McDFR1-Ra2* promoter sequence determined whether or not MYB10 binds to the *McDFR1* promoter, and that the MYBGAHV element may be an important *cis*-element in regulating *DFR1* expression by *MYB10*. Meanwhile, we also analyzed the potential bHLH or WD40 binding sites in F4 fragment, the results showed that F4 fragment lack related transcription factors binding sites. Hence, we speculate that *McDFR1-Ra1* promoter was preferentially used in ‘Radiant’ to make the leaf present red color in early development stages. And the *McDFR1-Ra2* promoter was keep low activity in ‘Radiant’ during leaf development.

PCR analysis showed that both the R6 and R1 promoters of *McMYB10* were present in the genome simultaneously in spring-red crabapple cultivars,^33^ and the same phenomenon (different promoters of one gene exist in one cultivars) was observed in the *McDFR1* promoter in spring-red crabapple cultivars. The *McDFR1-Ra2* promoter, which contains the important repressive F4 fragment and the *McDFR1-Ra1* promoter, which is strongly bound by McMYB10, are both present in the spring-red crabapple cultivar ‘Radiant’. We therefore deduce that the key anthocyanin-related genes contain activated and non-activated promoters simultaneously, and this may be a characteristic of the genomes of cultivars where red and green leaf color co-exist.

Alteration of the expression of *DFR* in plants represents an excellent strategy to modify the content of anthocyanins and flavonoids. In this regard, a suitable promoter is critical for the expression of exogenous genes, and our data suggest that the F4 fragment and the MYBGAHV *cis*-element may be used as valuable tools for the regulation of gene expression.

## Figures and Tables

**Table 1:** MYB-binding elements analysis of five promoters of *McDFR1*

*McDFR1-F1*		*McDFR1-F2*		*McDFR1-R*		*McDFR1-Ra1*		*McDFR1-Ra2*	
*Site name*	*Lot.*	*Site name*	*Lot.*	*Site name*	*Lot.*	*Site name*	*Lot.*	*Site name*	*Location*
—	—	—	—	MYB1AT	−1410	—	—	MYB1AT	−1614
MYB1AT	−966	MYB1AT	−831	MYB1AT	−965	—	—	MYB1AT	−1067
MYB1AT	−847	MYB1AT	−712	MYB1AT	−846	MYB1AT	−846	MYB1AT	−948
—	—	—	—	—	—	—	—	MYB1AT	−794
—	—	MYB1LEPR	−635	—	—	—	—	MYB1LEPR	−769
—	—	—	—	—	—	—	—	MYB1LEPR	−636
MYB2AT	−706	MYB2AT	−573	MYB2AT	−705	MYB2AT	−707	MYB2AT	−574
MYB2CONSENSUSAT	−706	MYB2CONSENSUSAT	−573	MYB2CONSENSUSAT	−705	MYB2CONSENSUSAT	−707	MYB2CONSENSUSAT	−574
MYB2CONSENSUSAT	−224	MYB2CONSENSUSAT	−224	MYB2CONSENSUSAT	−224	MYB2CONSENSUSAT	−225	MYB2CONSENSUSAT	−225
MYBATRD22	−847	MYBATRD22	−712	MYBATRD22	−846	MYBATRD22	−846	MYBATRD22	−948
MYBCORE	−706	MYBCORE	−573	MYBCORE	−705	MYBCORE	−707	MYBCORE	−574
MYBCORE	−224	MYBCORE	−224	MYBCORE	−224	MYBCORE	−225	MYBCORE	−225
MYBCORE	−227	MYBCORE	−227	MYBCORE	−227	MYBCORE	−228	MYBCORE	−228
MYBCOREATCYCB1	−224	MYBCOREATCYCB1	−224	MYBCOREATCYCB1	−224	MYBCOREATCYCB1	−225	MYBCOREATCYCB1	−225
MYBGAHV	−275	MYBGAHV	−275	MYBGAHV	−275	MYBGAHV	−276	—	—
MYBPZM	−880	MYBPZM	−745	MYBPZM	−879	MYBPZM	−879	MYBPZM	−981
—	—	—	—	MYBST1	−1549	—	—	—	—
—	—	MYBST1	−893	MYBST1	−1027	—	—	MYBST1	−1129
MYBST1	−827	MYBST1	−692	MYBST1	−826	MYBST1	−826	MYBST1	−928
