# Supplementary material for: Characteristics of dihydroflavonol 4-reductase gene promoters from different leaf colored Malus crabapple cultivars
Source: Hortic Res. 2017 Dec 13;4:17070–. doi: 10.1038/hortres.2017.70 (PMC5727492; doi:10.1038/hortres.2017.70)
Supplement: Supplementary Tables [file hortres201770-s1.docx]

**Supplemental Table S1.** Primer sequences used in this study.

| **ID** | **Sequence (5'-3')** | **Purpose** |
| --- | --- | --- |
| LAD1 | ACGATGGACTCCAGAGCGGCCGCVNVNNNGGAA | *McDFR1* promoter cloning |
| LAD2 | ACGATGGACTCCAGAGCGGCCGCBNBNNNGGTT |  |
| LAD3 | ACGATGGACTCCAGAGCGGCCGCHNVNNNCCAC |  |
| LAD4 | ACGATGGACTCCAGAGCGGCCGCVVNVNNNCCAA |  |
| LAD5 | ACGATGGACTCCAGAGCGGCCGCBDNBNNNCGGT |  |
| AC1 | ACGATGGACTCCAGAG |  |
| McDFR1-promoetr1 | TGCTCACGCCTGCTTTCTCTGTTTATCTCA |  |
| McDFR1-promoetr2 | TTCAAAAGTAACGCCCGAAGTCCCC |  |
| McDFR1-promoetr3 | ATAAAGTCACGCCAAGCAACTAACCTCA |  |
| DFR1promoter-Flame-up | GTTTTAGGTTTGAATCTCGTGAATGGAGA |  |
| DFR1promoter-Royalty-up | GTTGTACTGGTGGATAGGCCTTAATCT |  |
| DFR1promoter-Radiant-up | AGAAGTTTTAGGTTTGAATCTCGTGAATG |  |
| DFR1promoter-down | ATCTTGTGTGTATGTGCTTGCCGAA |  |
| pMD19-T-M13-F | GTAAAACGACGGCCAGTGAATTCG |  |
| pMD19-T-M13-R | CAGGAAACAGCTATGACCATGATTACG |  |
| SqRT-McDFR1F402 | GGCCGTTACATTTGTTCGTCAC | RT-PCR |
| SqRT-McDFR1R402 | TAAATCGAAACAGCATTTTATTTGAA |  |
| SqRT-McDFR2F422 | GGCCGTTACATTTGTTCGTCAC |  |
| SqRT-McDFR2R422 | AAAAGCACAATCAAACGAGCCCTA |  |
| SqRT-McMYB10-F | CAGGTGGTCATTGATTGCTAGA |  |
| SqRT-McMYB10-R | TCCAAAGGTCCGTGCTAAAG |  |
| qRT-McDFR1-F | CACACGCACAATAGTTGAGGTTA | qRT-PCR |
| qRT-McDFR1-R | TTTCAAGTCATGCAATCAAGAGT |  |
| qRT-McDFR2-F | CTGAGCAGCATACGCCCTCTTTTGG |  |
| qRT-McDFR2-R | ATCTTCTGTTGAATCTGTGTACGTG |  |
| qRT-McMYB10-F | GGACCAGCAGCAGGAAACTA |  |
| qRT-McMYB10-R | ACAACCCTCCATTAATGCCGAC |  |
| pLacZi-F | CCTGTGATATTATCCCATTCCATGCG | Yeast one-hybrid assay |
| pLacZi-R | AGCTGCATTAATGAATCGGCCAA |  |
| McDFR1 promoter  -XhoI | CCGCTCGAGAGAAGTTTTAGGTTTGAATC  TCGTGAATG |  |
| pB42AD-F | CATGAAATTGAAGCGGATGTTAACGAT |  |
| pB42AD-R | CAAACCTCTGGCGAAGAAGTCCAA |  |
| McMYB10-F-BamHI | CGCGGATCCATGGAGGGATATAACG |  |
| McMYB10-R-SalI | GGCGTCGACTAGGTATTCTTCTTTTG |  |
| McMYB10-F-XbaI | GCTCTAGAATAAGAGATGGAGGGATATAACGA | MYB10 protein obtained |
| McMYB10-F-HindIII | CCAAGCTTGATGGTACCACAGAAGTGAGAA |  |

**Supplemental Table S2.** Probe sequences used in this study.

| **Motif name** | **Probes (5’-3’)** |
| --- | --- |
| MYB1AT (WAACCA) | TTATTATTATCTATAACCATTACTATTTATC |
| MYBGAHV (TAACAAA) | GTTGAGCAGGTAACAAATGAAGTCAAG |
| MYB1LEPR (GTTAGTT) | CAGAATTGAAAAACTAACATTTTAATGTG |
| MYBST1 (GGATA) | AATATATTTATGGATAAATTATGATTA |

**Supplemental Table S3.** Correlation analysis of the expression level of *McDFR1* and *McDFR2* with the contents of anthocyanin and the transcript level of *McMYB10*

|  | **‘Royal’** | | **‘Radiant’** | | **‘Flame’** | |
| --- | --- | --- | --- | --- | --- | --- |
|  | *McDFR1* | *McDFR2* | *McDFR1* | *McDFR2* | *McDFR1* | *McDFR2* |
| Anthocyanin | 0.718* | 0.253 | 0.735 * | 0.088 | 0 | 0 |
| *McMYB10* | 0.960** | 0.521 | 0.723* | 0.654 | 0.276 | -0.621 |

*, Correlation coefficient was significant at the *P* ≤ 0.05 level; **, Correlation coefficient was significant at the *P* ≤ 0.01 level.
